# Asphalt Binder Modification with Hazelnut and Walnut Shells as Valued Antioxidant Sources: Effects on Rheological and Main Physicochemical Post-Oxidation Indicators

**DOI:** 10.3390/ma19081560

**Published:** 2026-04-14

**Authors:** Carlos Manterola-Barroso, Karina Godoy-Sánchez, Erick Scheuermann, Ivanka Netinger Grubeša, Dunja Šamec, Cristian Meriño-Gergichevich

**Affiliations:** 1 Doctoral Program in Science of Natural Resources, Universidad de La Frontera, Temuco 4811230, Chile; carlosignacio.manterola@ufrontera.cl; 2 Scientific and Technological Bioresources Nucleus (BIOREN-UFRO), Universidad de La Frontera, Temuco 4811230, Chile; karina.godoy@ufrontera.cl (K.G.-S.); ericks@ufrontera.cl (E.S.); 3 Laboratory of Physiology and Plant Nutrition for Fruit Trees, Faculty of Agricultural Sciences and Environment, Universidad de La Frontera, Temuco 4811230, Chile; 4 Laboratory of Soil Fertility, Faculty of Agricultural Sciences and Environment, Universidad de La Frontera, Temuco 4811230, Chile; 5 Chemical Engineering Department and CIBAMA, Faculty of Engineering and Science, Universidad de La Frontera, Temuco 4811230, Chile; 6 Department of Construction, University North, 42000 Varaždin, Croatia; inetinger@unin.hr; 7 Department of Food Technology, University North, 48000 Koprivnica, Croatia; dsamec@unin.hr; 8 Department of Agricultural Production, Faculty of Agricultural Sciences and Environment, Universidad de La Frontera, Temuco 4811230, Chile; 9 Program of Master in Fruitculture, Faculty of Agricultural Sciences and Environment, Universidad de La Frontera, Temuco 4811230, Chile

**Keywords:** asphalt binder, nutshell phenolics, antioxidant modification, oxidative aging

## Abstract

Oxidative aging drives asphalt pavement degradation, causing critical structural failures. This study evaluated hazelnut (HS) and walnut shell (WS) powders (0–3% *w*/*w*; 10–12 μm) as sustainable antioxidants, from valued residues, to mitigate thermo-oxidative aging in CA-24 binders. After evaluating the antioxidant potential (ORAC; Oxygen radical absorbance capacity, and TPC; Total phenolic content), modified binders underwent RTFO (Rolling thin film oven) and PAV (Pressure aging vessel) aging, evaluated by Fraass fragility, Relative Aging Index (RAI), dynamic shear rheometry (G*/sin δ), and Multiple Stress Creep Recovery (MSCR). WS exhibited significantly higher antioxidant capacity (6000 μmol TE g DW^−1^) and TPC than HS. The 3% treatments demonstrated optimal antioxidant efficacy, reducing long-term RAI by 14% and improving low-temperature flexibility by 3.8 °C (Fraass point −12.3 °C). However, MSCR revealed initial plasticizing effects decreasing elastic recovery (70%) and increasing non-recoverable compliance (Jnr) compromising unaged rutting resistance. Principal component analysis confirmed progressive diversification of aging-induced properties, evidencing complex multivariate trajectories. Ultimately, while nutshell derived phenolic modifiers provide effective concentration-dependent antioxidant protection, practical application requires optimization through targeted phenolic extraction, particle engineering, or elastomeric co-modification. Balancing aging resistance with high temperature stability remains essential for advancing these sustainable biomodification strategies in road infrastructure.

## 1. Introduction

Nowadays, around 95% pavements worldwide contain asphalt binders (AB) [1]. In the U.S., more than 90% of road surfaces are made of asphalt. In fact, 1.6 trillion tons (t) of this material has been produced for road construction worldwide in the last decade [2]. The specific market of the AB industry was valued at over USD 60 billion in 2023–2024, with Asia–Pacific being the leading region in road infrastructure investment [3]. However, the industry is currently facing critical challenges, mainly due to the oxidative aging of ABs, an irreversible process characterized by complex changes in the chemical composition of the binder [4,5]. It is triggered by the formation of carbonyl compounds and sulfoxides that cause hardening and loss of flexibility [6], becoming brittle and susceptible to cracks and structural failures [4,7,8,9]. This issue represents one of the main mechanisms involved in asphalt pavement degradation, causing substantial economic losses that can reach between 1.5% and 2% of GDP for the construction and repair of road infrastructure [10].

Oxidative aging (OA) mechanisms include oxygen diffusion, free radical formation, and condensation reactions that disturb the balance between saturated, aromatic, resinous, and asphaltene fractions [5,11,12], significantly compromising the durability and functionality of road infrastructure [5,10]. In fact, in the last few decades, road technology research and efforts have been focused on the incorporation of asphalt technology into products and processes in order to improve AB properties directed to modulate the OA throughout the AB lifespan [9,13]. Therefore, the application and/or incorporation by modification of Aox additives in ABs has become a promising strategy to mitigate these effects, as they act primarily as free radical scavengers that interrupt oxidation chain reactions and preserve the rheological properties of the binder [14,15,16,17]. This opens an opportunity to focus research on the development of sustainable AB antioxidant additives [13]. In fact, the AB additives market was valued at $4.62 billion in 2024 and is estimated to reach $6.6 billion by 2030 (CAGR 7.4%) [3], showing stable growth. Thus, there is a relevant commercial and environmental framework for the generation of sustainable Aox additives, which could to modulate OA processes in ABs in an environmentally friendly development pathway [18,19,20]. In recent years, several authors have reported advances in the incorporation of agro-waste rich in phenolics, mainly from lignocellulosic resources (rich in phenolic compounds), in order to mitigate the OA in asphalt matrixes.

Polyphenols, sometimes called phenolics, are plant-specialized metabolites known for their antioxidant activity. They are often present in high concentrations in non-edible plant parts; therefore, this agricultural waste represents a cheap and readily available source of antioxidants. Sun et al. (2025) [21] investigated the mechanism of lignin to act as an antioxidant in AB, demonstrating how lignin improves the high-temperature rheological properties of asphalt and delays OA. In addition, Yang et al. (2022) [22] demonstrated that catechins block asphalt aging at a chemical level by providing active hydrogen atoms to react with O_2_ and free radicals in AB matrixes. Moreover, Park et al. (2022) and Pahlavan (2022) [23,24] showed the effectiveness of used phenolic-rich bio-oil, which can interact with free radicals generated during oxidative aging. Phenolic compounds from lignocellulosic sources can neutralize free radicals by donating hydrogen, resulting in phenoxyl radicals that act as free radical scavengers [23]. Among the lignocellulosic agro-waste produced worldwide, nutshells (NS) stand out due to their chemical composition, antioxidant capacity, and microstructure and physicochemical features [25]. In this sense, the nut industry (main industry) has grown over the past decade, with production around 7.7 million t in 2023/24. Hazelnut (*Corylus avellana* L) and walnut (*Juglans regia* L) represented 48% and 15.5% respectively [26,27], which was confirmed during 2023/24 with production over 4.8 million t [25,27]. Currently, the main nut residue corresponds to their shell, which represents over 55% of the total whole nut biomass weight, resulting in a huge raw material availability [25]. Nevertheless, those shells are not reused at an industrial or semi-industrial scale due to a huge gap in environmental awareness and technological capacity to transform them into useful by-products [18,28]. Nowadays, they are mainly used as fuel for heating, which results in the generation of associated emissions, conferring low added value without taking into account their interesting properties [29,30]. These shells have exhibited similar microstructural, chemical, antioxidant and physicochemical properties, highlighted by a heterogeneous shape and useful microstructural features [25,31].

The main chemical composition is around 46.6% holocellulose, 25.4% α-cellulose, and between 29.9 and 49.1% lignin [18,32,33,34]. The Aox capacity of hazelnut shells (HS) has been reported by several authors as around 1800 to 5000 μM Teq (Trolox equivalents) g^−1^ in addition to significant total phenolic compound content, equivalent to 394. 2 ± 15.8 mg GAE (Gallic acid equivalents) g^−1^ [25,35,36,37]. Similarly, the phenolic content and antioxidant capacity of walnut shells (WS) were determined by Queirós et al. (2020) [32], with 31.79 mg GAE g DW (Dry weight)^−1^ and TEAC (Trolox Equivalent Antioxidant Capacity) values around 85 18.86 mg TE g DW^−1^, respectively. Therefore, NSs have been shown as promising lignocellulosic raw materials for testing as antioxidant agents against OA processes.

This research represents an improvement in road construction materials, based on the hypothesis that an antioxidant asphalt modifier developed from HS and WS can significantly reduce oxidative damage in asphalt binders, extending the useful life of pavements and contributing to the circular economy. Moreover, this study aimed to comprehensively evaluate the physicochemical and antioxidant properties of this modifier, as well as its impact on the rheological, chemical, and aging properties of modified asphalt binders, developing a sustainable, economically viable, and technically superior technological solution for the road paving industry.

## 2. Materials and Methods

All conducted methodologies were based on Chilean and international standards adopted by the Ministry of Public Works (MOP) and validated by the National Road Laboratory (vialidad.mop.gob.cl), ensuring that the results are applicable for implementation in national road works (Manual de Carreteras 2019 [38], AASHTO, MC, ASTM and Nch standards). Analytical triplicates, blanks (TPC and ORAC), and certified standards were followed in order to validate the accuracy and reproducibility of the obtained results.

### 2.1. Nutshells, Asphalt Binder and Modifier Development

HS and WS samples were obtained during the 2019–2020 harvest season, from two different orchards located in southern Chile (La Araucanía region): Radal (39°10′06′′ S, 72°19′11′′ W; Altitude: 168 m.a.s.l.) and “Campo Experimental Maquehue” (38°50′28.5′′ S, 72°41′40.4′′ W; Altitude: 200 m.a.s.l.), respectively. Nutshell samples underwent an optimized preparation process: (1) manual cleaning with double-distillated (dd) H_2_O and sorting to remove impurities, (2) controlled drying/stabilization process at 40 °C (72 h in a forced-air oven (Heratherm OGS100, Thermo Scientific, Waltham, MA, USA; Memmert UF-55, Büchenbach, Germany)) until a moisture content of 0.1% (*w*/*w*) (until constant mass), subsequently determined by a Bel Engineering (I-Thermo G163L) thermo balance (Monza, Italy), and (3) initial mechanical grinding and particle size reduction (10–12 μm) using a Retsch (ZM 200) ultra-centrifugal mill (Haan, Germany).

Bituminous material, a conventional CA-24 (DYNAL S.A, Santiago, Chile) AB (Batch 1765, 463-A) was also used. This grade of binder was selected for its representativeness in national road applications and its compatibility with the climatic conditions of the studied area, and was equivalent to PG 64-22 according to the Superpave classification. The basic properties of the binder were characterized before modification to establish reference values (Table 1).

### 2.2. Nutshells’ Antioxidant Capacity and Total Phenolic Content

The antioxidant capacity (AC) of the prepared modifier was evaluated using multiple complementary methods. ORAC (Oxygen Radical Absorbance Capacity) was determined following the method proposed by Manterola-Barroso et al. (2022) and Gavilán-CuiCui et al. (2024) [35,36] (Nº 2021-A-8614), registered in the Chilean Department of Intellectual Rights (DDI), and adapted for WS samples. Analyses were measured with a Biotek Synergy H1 Hybrid multi-mode reader (Winooski, VT, USA) and data was interpreted by Gen5™ V 2.0 software. In order to complement the obtained ORAC data, total phenolic content (TPC) analyses were conducted following the Folin–Ciocalteu method based on a calibration curve between 0 and 750 µg mL^−1^, measured by a TECAN Infinite^®^ 200 PRO NanoQuant multi-plate reader (Männedorf, Switzerland) at a wavelength of 765 nm [35]. ORAC results were expressed in Trolox equivalent (TE) units (μM TE g DW^−1^), while TPC was expressed in gallic acid equivalents (GAE) (mg GAE g DW^−1^).

### 2.3. Asphalt Binder Modification

Four modified asphalt formulations were prepared for each modifier (HS and WS), with different concentrations of the antioxidant modifier (0% or control, 1, 2, and 3%, *w*/*w*), following the method proposed by Calabi-Floody et al. (2012) [40]. Each AB sample was heated to 95 °C in order to obtain viscosities ranging between 7000 and 8000 P, allowing for a homogeneous mixture. Subsequently, HS and WS modifiers were added separately in powder. Each mixture was then homogenized until uniformity was achieved. The same process was performed for each control sample, without adding Aox, to maintain the exposure and “thermal history” of each sample.

### 2.4. Forced Aging Processes

Firstly, short-term aging was performed using the rolling thin film oven test (RTFOT) method in accordance with AASHTO T-240 [41]. Briefly, 35 g of each sample was exposed to 163 °C for 85 min under continuous air flow, simulating the aging conditions during the production and placement of asphalt mixtures. Subsequently, the samples previously aged by RTFOT were exposed to long-term forced aging, which was performed using a 9500 Prentex oven (Sunnyvale, TX, USA) for the pressure aging vessel (PAV) method, following AASHTO R-28 [42]. Samples were exposed to 100 °C for 20 h under an air pressure of 2.07 MPa, followed by vacuum degassing to simulate 5–10 years of aging in service.

Finally, all forced-aged samples were subjected to chemical, rheological and physical analyses in order to evaluate each addition % and used NS effect on AB main properties and post-oxidation indicators.

### 2.5. Rotational Viscosity and Fraass Point

In order to evaluate the effects on viscoelastic properties at medium-high temperatures, rotational viscosity at 60 °C ± 1 °C was determined by a Brookfield (Stoughton, MA, USA) DV-III ULTRA digital rheometer. Results were interpreted as a Relative Aging Index (RAI), which was described as a difference between two different aging times. RAI was calculated, for each combination of aging times, as follows:

(1) $$RAI\;=\;\frac{\left(\eta \mathrm{Aging}\;\mathrm{state}-\eta \mathrm{Original}\;\mathrm{state}\right)}{\eta \mathrm{Original}\;\mathrm{state}}$$

Low-temperature properties were evaluated by Fraass break point, which was determined for each sample at each aging stage using a Celestron IP42C instrument (Santiago, Chile). Moreover, all determinations were based on the MC 8.302.24 method.

### 2.6. Dynamic Shear Rheometer (DSR) and Multiple Stress Creep Recovery (MSCR)

Rheological characterization was performed using an Anton Paar (Graz, Austria) dynamic shear rheometer according to the AASHTO T-315 standard [43]. Measurements were conducted on unaged, post-RTFOT, and post-PAV samples using parallel plate geometry (25 mm diameter, 1 mm thickness for temperatures ≥ 46 °C; 8 mm diameter, 2 mm thickness for temperatures between 4 and 40 °C). The complex shear modulus (G*), phase angle δ, and derived parameters were determined to evaluate resistance to rutting, fatigue, and thermal cracking.

To evaluate the AB samples’ resistance to permanent deformation under cyclic loads, several MSCR test were performed utilizing an Anton Paar (Graz, Austria) SmartPave 102 modular compact rheometer, according to AASHTO T-350 [44]. Analyses were conducted at 64 °C by applying stresses of 0.1 and 3.2 kPa in cycles of 1 s of loading and 9 s of recovery, determining the percentage of recovery (%R) and non-recoverable compliance (J_nr_).

### 2.7. Experimental Design and Statistical Analyses

To determinate the effect of HS and WS as antioxidant modifier agents on the main rheological, physical and chemical properties of control aging (C), post-RTFO (ST) and post-RTFO + PAV (LT) AB modified samples, a completely randomized design was performed. It was based on two NS (HS and WS) modifier agents, four addition treatments (0, 1, 2, and 3%) and three forced aging times (C, ST, LT). All statistical analyses were conducted in triplicate and reported as mean ± standard deviation (SD). Two-Way ANOVA was carried out and their main interactions were determined for all evaluated rheological and physical parameters, while a One-Way ANOVA was conducted for ORAC and TPC determinations as well as a Pearson correlation test. Both analyses of variance were followed by Tukey’s and Duncan’s multiple comparison tests, respectively. Moreover, in order to understand the closeness and interaction behavior among all rheological and determined dependent variables, a principal component analysis (PCA) was performed. All mentioned statistical analyses were carried out in at least triplicate to ensure statistical reproducibility, and the free software R^©^, V 3.6.1 (R Development Core Team, Vienna, Austria) was used.

## 3. Results

### 3.1. Nutshell Modifiers’ Antioxidant Properties

**Figure 1:**
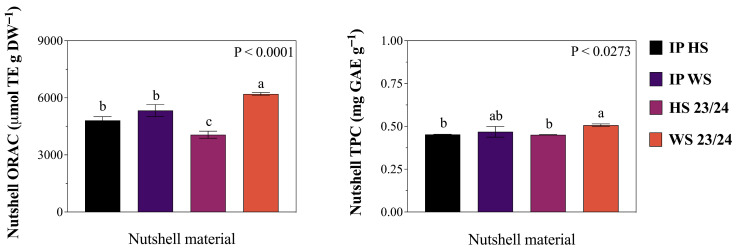
ORAC antioxidant capacity (μmol TE g DW^−1^) and total phenolic content (μg GAE g DW^−1^) of 2023/24 season HS and WS (Tonda di Giffoni, Franquette), and of HS and WS industrial pool (IP) shell samples. All samples came from La Araucanía region commercial orchards. Bars represent the average of three replicates ± S.E, while different lower case letters indicate statistical differences (*p* ≤ 0.05) between all evaluated nutshell samples.

The comparative analysis of ORAC antioxidant capacity and phenolic content (TPC) between hazelnut shell (HS) and walnut shell (WS) modifiers revealed significant differences across different sources and nutshell materials (Figure 1). The ORAC antioxidant capacity demonstrated statistically significant differences (*p* ≤ 0.05) among the evaluated nutshell samples; WS (Franquette variety, 2023/24 season) reached the highest antioxidant capacity (6000 μmol TE g DW^−1^), followed by its industrial pool (IPWS), which was around 5000 μmol TE g DW^−1^. The industrial pool (IP) samples showed intermediate values as well as HS from the 2023/24 season. In fact, IPHS exhibited around 4500 μmol TE g DW^−1^ and IPWS over 5000 μmol TE g DW^−1^, although non-significant differences were found among both IP nutshell samples.

TPC determination supported the ORAC findings. In fact, WS 2023/24 samples achieved the maximum determined phenolic content (0.506 mg GAE g DW^−1^), around 7.5% higher than industrial pool material (IPWS), which exhibited 0.468 mg GAE g DW^−1^. Conversely, both HS 2023/24 and IPHS TPC were significantly lower than both WS samples (*p* ≤ 0.05). Indeed, there was determined a 7.8% lower TPC on HS samples in comparison to WS evaluated samples (means). Statistical analysis confirmed that both WS evaluated samples (IP and 2023/24 season) consistently displayed higher ORAC antioxidant capacity and TPC compared to HS across all examined samples. A strong correlation between ORAC values and TPC (r^2^ > 0.928) was found; these results suggest that phenolic compounds may constitute a main role in the antioxidant capacity in both NS samples.

### 3.2. Low- and High-Temperature Effects

The Fraass fragility point determination revealed highly significant (*p* < 0.0001) effects of HS and WS, in both control and short-term aging, on low temperature performance. Moreover, it was demonstrated that the superior low-temperature performance enhancement was achieved through NS modification. Control samples exhibited break temperatures of −8.5 °C, while the incorporation of 3% HS modifier significantly improved low-temperature flexibility, reducing the Fraass value to −12.3 °C throughout all aging periods.

**Figure 2:**
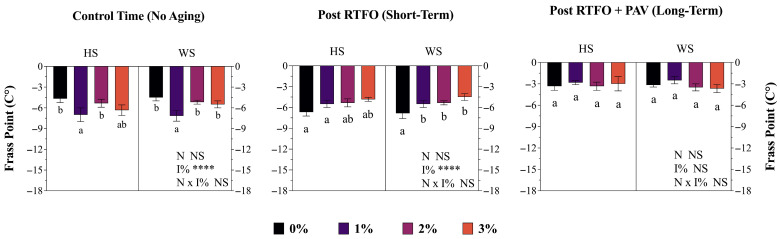
Modified asphalt binder (AB) Fraass fragility point (°C) throughout three different time periods (Control time, short-term, and long-term aging). HS corresponds to hazelnut shell and WS to walnut shell. Each point represents the average of three replicates ± S.E. Different letters indicate statistical differences (*p* ≤ 0.05) between the nutshells used (HS and WS) in the same locality. Two-way ANOVA results are shown as follows: **** *p* < 0.0001, NS = non-significant.

Finally, these results showed that HS and WS had different effects on the aging resistance of the modified asphalt binder. The Fraass point showed a significant improvement in low-temperature flexibility with both additives at 1% of addition, reaching values of −7.0 °C for HS and −7.1 °C for WS in the control condition, compared to −4.8 °C and −4.9 °C for the unmodified binder, respectively. This improvement was partially maintained after long-term aging (PAV), where binders modified with 1% HS and WS showed Fraass temperatures of −2.8 °C and −2.5 °C, versus −3.33 °C and −3.1 °C for the control (Figure 2).

### 3.3. Rutting Performance

**Figure 3:**
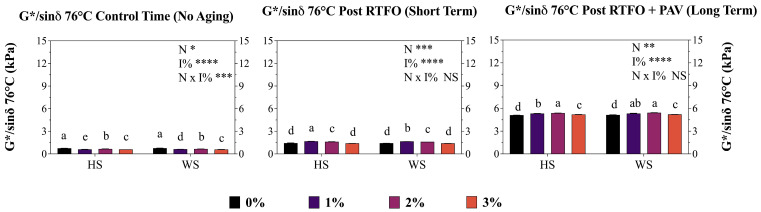
Modified asphalt binder (AB) rutting performance (g*sin*δ*) (kPa) at 76 °C. Results obtained for both studied nutshell modifiers and in three different periods (control time, short-term, and long-term). HS corresponds to hazelnut shell and WS to walnut shell. Each point represents the average of three replicates ± S.E. Different letters indicate statistical differences (*p* ≤ 0.05) between the nutshells used (HS and WS) in the same locality. Two-way ANOVA results are shown as follows: **** *p* < 0.0001, *** *p* < 0.001, ** *p* < 0.01, * *p* < 0.05, and NS is non-significant.

The G*/sinδ parameter corresponds to the standard rheological index that quantifies the rutting resistance of an asphalt binder under high temperature conditions, in accordance with the Superpave specification. For asphalts modified with antioxidant additives, this parameter indicates the binder’s capacity to maintain its stiffness and elastic behavior, even after oxidative aging, which translates into higher durability and lower permanent deformation in service [14]. Results showed that under control aging conditions (no aging), a highly significant interaction (*p* < 0.001) between nutshell material (N) and incorporation % (I%) was determined (N × I%). In fact, concentrations of 1% and 3% produced significant reductions on the evaluated parameter; HS 1% exhibited a decrease of 19.8%, while 3% showed a reduction of 20.2%, in comparison to the addition control (0%). Similarly, WS 1% and WS 3% exhibited a decrease of 18.9% and 22.1%, respectively, on the rutting parameter. Subsequently, post-RTFO aging (short-term), a non-significant interaction was determined between N and I% factors (N × I%). Those factors exhibited highly significant values that demonstrated the negative effect of the nutshells used and incorporation % on G*/sinδ in the short-term aging time. In fact, a negative rutting performance was observed for 1% and 2% (both HS and WS) treatments. Nevertheless, HS and WS 3% exhibited non-significant results in comparison to the short-term control sample, which indicates that non-negative effects on rutting parameter were generated by both evaluated nutshells at a 3% of addition. Likewise, after PAV aging (long-term), the same variations were determined in HS 1 and HS 2% treatments, and also in WS 1 and WS 2% (Figure 3). This phenomenon suggests that the microstructural characteristics and particle size of nutshells may interfere with the structural strength under moderate thermal aging conditions.

Finally, after RTFO and PAV aging (short and long term), all treatments exhibited moderate to large increases in G*/sinδ (1.4–5.5%) compared to their respective controls, indicating a negative effect on the rheological properties’ preservation at high temperatures (76 °C).

### 3.4. Viscosity-Based Relative Aging Index (RAI)

Viscosity at 60 °C showed a significant (*p* < 0.005) reduction with the addition of both NSs at the control aging time, which indicates a possible initial plasticizing effect.

**Figure 4:**
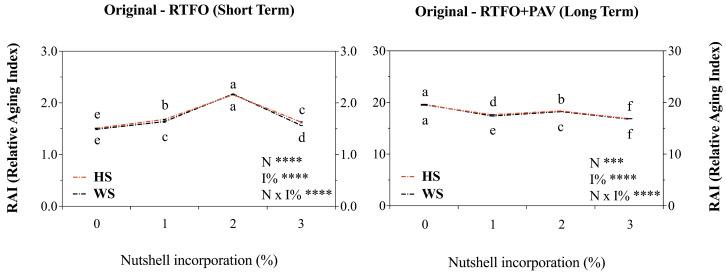
Modified asphalt binder (AB) rotational viscosity at 60 °C (59–61 °C), interpreted as relative aging index (RAI) throughout three different periods (control time, short-term, and long-term forced aging). HS corresponds to hazelnut shell and WS to walnut shell. Each point represents the average of three replicates ± S.E. Different letters indicate statistical differences (*p* ≤ 0.05) between the nutshells used (HS and WS) in the same locality. Two-way ANOVA results are shown as follows: **** *p* < 0.0001, *** *p* < 0.001.

In both cases (HS and WS), the trend in viscosity variation oscillated. Significant gradual reductions in viscosity values were observed in both the control (no aging) and long-term (post-PAV) times. However, in the short term (post-RTFO), viscosity increased by up to 26.6% (for HS and WS) in 2% additions. The Relative Aging Index (RAI), based on previously described rotational viscosity values, revealed distinguishable patterns that confirm differentiated effects according to the aging stages (Figure 4). Statistical analysis confirmed highly significant main effects of NS (*p* < 0.0001) and aging stage (*p* < 0.001), with significant interaction effects (*p* < 0.001) indicating differential behavior against oxidative hardness among forced aging stages and added NS. After short-term aging (control to short term), RAI values showed a tendency to increase significantly after 1% addition (11.3%) and 2% addition (42.9%) compared to the control. However, the 3% addition showed a drastic decrease in RAI compared to HS and WS 2% addition and was not significant, except with WS1%, compared to 1%. Likewise, a 7.7% increase in HS and WS at 3% RAI (compared to the control) was determined. However, in the long term, RAI values showed significant decreases for both HS and WS, added at 1% (10.8 and 11.3%), 2% (6.2 and 7%), and 3% (14 and 14.3%), compared to the control. These results confirm better performance of the added modifiers at 3% and only in the long term.

### 3.5. Multiple Stress Creep Recovery (MSCR)

**Figure 5:**
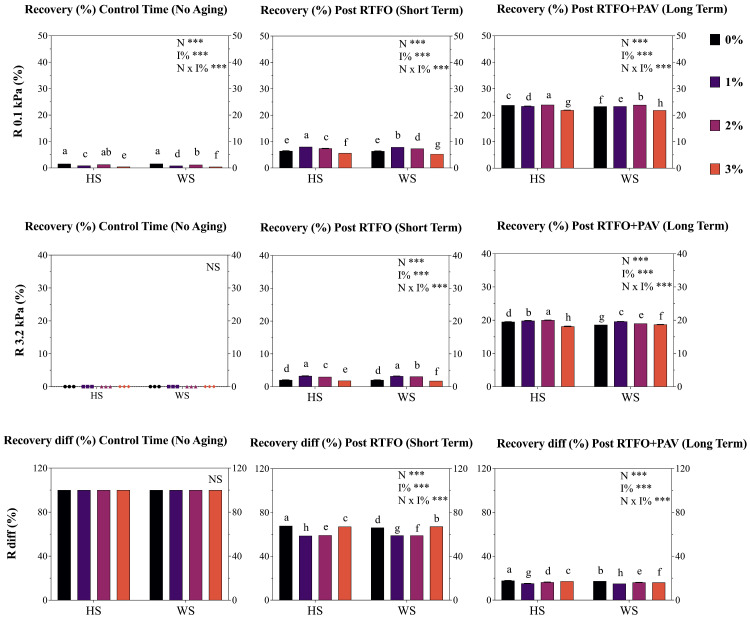
Modified asphalt binder (AB) multiple stress creep recovery (MSCR) recovery (R) at 0.1 and 3.2 kPa and difference (R diff %) (kPa^−1^) results for both studied nutshell modifiers. HS corresponds to hazelnut shell and WS to walnut shell. Each point represents the average of three replicates ± S.E. Different letters indicate statistical differences (*p* ≤ 0.05) between the nutshells used (HS and WS). Two-way ANOVA results are shown as follows: *** *p* < 0.001.

The MSCR analyses (Figure 5) evaluated stress-dependent viscoelastic recovery characteristics under standardized loading conditions, with highly significant effects (*p* < 0.001) for nutshell used (N), concentration (I%), and stress level (kPa), with significant interactions (*p* < 0.005) between all mentioned factors. At the 0.1 kPa stress level, no aged control AB samples exhibited 1.56% recovery, and the 1% treatment decreased by 46.0 and 48.1% for HS and WS, respectively. Conversely, the 2% results (both HS and WS) showed higher results than 1%, although they were 17.8 and 23% lower than the control. The same effect was determined on HS and WS 3%, which significantly (*p* < 0.001) reduced the recovery percentage by 69.8 and 71.1%, respectively. Short-term results (0.1 kPa) showed an increase in 1% in both HS and WS (20 and 18.8%). These results were similar, increasing 13.1 and 12.7% in comparison to the control. Conversely, the HS3% and WS3% treatments decreased the R percentage by 19.1 and 17.3%. For long-term aging, values slightly decreased for HS and WS with the 1 and 2% additions (1.51 to 2.3% mean), while 3% significantly decreased R values by 7.9 and 8.1% for HS and WS, respectively.

At the elevated stress level (3.2 kPa), which represents severe loading conditions, HS and WS showed an increase from R 0 (control time) in the different addition treatments, rising to values of 2.056 and 2.052 for the control (0%). This value increased by 37.3–34.2% (1% addition) and 31.5–33.8% (2% addition) in the short term. However, the 3% addition showed a significant decrease of 8.3% and 12.6% (for Hs and WS, respectively) compared to the control. In the long term, the behavior was similar to that in the short term, maintaining the upward trend in the R value for the 1% and 2% addition treatments for both HS and WS. These values increased by 1.5–5.0% (1% addition) and 2.4–2.6% (2% addition), respectively, although the 3% addition showed a decrease of 7.2–0.5% in the R value compared to the control (HS and WS, respectively).

Finally, the recovery difference parameter (R diff %) quantified stress sensitivity, with lower values indicating better performance consistency across stress levels. Therefore, the decreasing trend of this value among the aging stages was expected. HS modification maintained superior performance compared to WS in the short term, and exhibited the best performance with 1 and 2% additions, decreasing the control values from 2.2 to 10.9%, respectively. WS showed the same patterns but with lower values in comparison to its control. Similarly, the behavior shown at the long term in the different treatments of HS and WS followed the same pattern as that in short term. Values of 1, 2%, and 3% trended to decreased Rdiff %, with the increasing percentages ranging from 3.5 to 14.4% among the different evaluated treatments and samples.

Non-Recoverable Compliance Assessment J_nr_ parameters quantified permanent deformation resistance under creep loading conditions. Therefore, low J_nr_ values are preferred in applications requiring high rutting resistance (high-traffic routes, areas subject to high loads), while high J_nr_ values would be acceptable only in applications where load and temperature are not critical to material stability.

Highly significant effects (*p* < 0.001) for nutshell used (N), concentration (I%), and stress level (kPa) were revealed, with significant interactions (*p* < 0.005) between all mentioned factors. At the control time, the HS and WS treatments with 1 and 3% addition showed a higher J_nr_ value (*p* < 0.001), reflected in an increase of 23.3 and 20.6% for HS1% and WS1%, while in HS3% and WS3% an increase of 27.1 and 23.5% was determined. In the short term, the 1% and 2% treatments decreased by 22.7–20.2% and 18.1–3.1% (of HS and WS, respectively) compared to the control. Nevertheless, the 3% treatment results were slightly lower than the control (mean 1%), as well as for the long-term. Similarly, the 1% and 2% treatments showed the same behavior in the control and long-term time frames, evidencing different behavior from all treatments in the short term (with the understanding that it emulates oxidation similar to that at the time of its application in the field).

Elevated stress results (3.2 kPa) revealed a similar effect to those obtained at 0.1 kPa, with the same significant increase of 1% and 3% for HS and WS compared to the control. However, in the short term, these two treatments tended to show a significant decrease (*p* < 0.005) in the J_nr_ values compared to the control. This effect was determined to be slightly lower in the long term, where all treatments showed values inferior to the control, thereby demonstrating a lower susceptibility to deformation.

**Figure 6:**
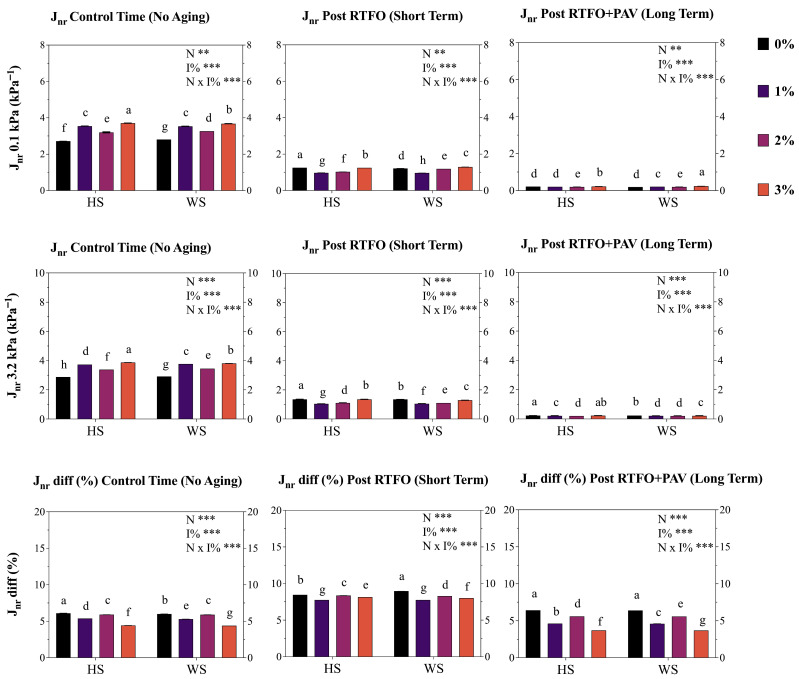
Modified asphalt binder (AB) multiple stress creep recovery (MSCR) J_nr_ parameters at 0.1 and 3.2 kPa, and difference percentage (%) (kPa^−1^) for both studied nutshell modifiers. HS corresponds to hazelnut shell and WS to walnut shell. Each point represents the average of three replicates ± S.E. Different letters indicate statistical differences (*p* ≤ 0.05) between the nutshells used (HS and WS). Two-way ANOVA results are shown as follows: ** *p* < 0.005, *** *p* < 0.001.

Finally, the J_nr_ difference percentage (J_nr_ diff%) quantified stress sensitivity, showing the same pattern across all aging stages. A significant decrease in the control time was exhibited, as well as in the short term, in all treatments (1, 2 and 3%) compared to the control (Figure 6), indicating potential superior stress-independent behavior.

**Figure 7:**
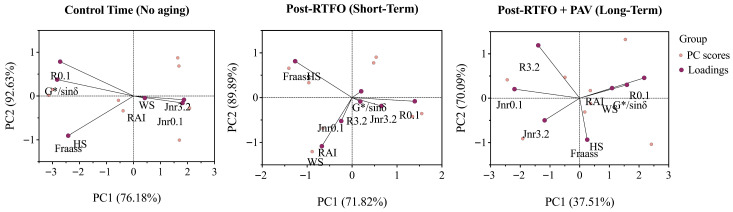
Principal components (PCs) for the experimental variables determined in the modified and forced-aged AB. The principal component analysis (PCA) biplot shows the distribution of the experimental variables according to PCs and grouped according to the different forced-aged period of time (control time, short-term and long-term). A 95% threshold was considered for the PCA and the analyses were carried out considering all the response variables determined during this study.

Physical, mechanical, and rheological effects after the addition of different percentages (0–3%) of HS and WS at the three different forced aging times (control; short-term; long-term) were determined for the response variables previously described (Frass point, RAI, MSCR data, and G*/sinδ). The distribution of these variables and the relationship between each of them, at the different aging stages, was determined using a principal component analysis (PCA). The obtained results showed how the experimental variables (Fraass point, RAI, MSCR results, and G*/sinδ) were grouped and distributed differently depending on the time and type of aging, indicating that the properties of the modified AB underwent significant transformations with the progression of aging (Figure 7).

The principal component analysis conducted at the 95% confidence threshold revealed critical insights into the multivariate relationships between experimental variables across aging stages. The variance explained by PC1 decreased from 76.2% (control time) to 37.51% (long-term PAV aging), while PC2 increased from 12.0 to 30.3%, indicating a progressive diversification of property changes during oxidative aging. This variance redistribution suggests that oxidative aging induces complex, multidirectional transformations in asphalt properties that cannot be captured by single-parameter assessments, supporting the necessity of comprehensive characterization protocols that integrate rheological, chemical, and physical parameters [45,46].

The biplot distribution patterns demonstrate that variables such as Fraass point, RAI, MSCR parameters, and G*/sin δ cluster differently according to aging stage, confirming that nutshell modification alters the fundamental aging trajectory of asphalt binders. This multivariate behavior aligns with the complex oxidation mechanisms described by Cui and Wang (2024) and Hu et al. (2023) [47,48] through molecular dynamics simulations, where oxidative aging generates concurrent changes in molecular weight distribution, polar functional group formation, and colloidal structure reorganization that manifest as coordinated shifts across multiple performance parameters.

## 4. Discussion

### 4.1. Nutshell Modifiers’ Antioxidant Properties

The differential antioxidant capacity and total phenolic content (TPC) determined between hazelnut shell (HS) and walnut shell (WS) modifiers constitute a fundamental baseline for understanding their potential performance as antioxidant agents in asphalt binders. WS samples exhibited significantly higher ORAC values (up to 6000 μmol TE g DW^−1^) and TPC (0.506 mg GAE g DW^−1^) compared to HS counterparts, which aligns with previous findings reported by Queirós et al. (2020) and Manterola-Barroso et al. (2022 and 2025) [25,32,35], who documented HS and WS antioxidant capacities which ranged from 1800 to 5000 μM EqT g^−1^, and significant phenolic content in WS compared to HS. The strong correlation (r^2^ > 0.928) between ORAC and TPC values confirms that phenolic compounds are the principal contributors to antioxidant activity in both nutshell materials, consistent with mechanisms described by Yang et al. (2022) and Park et al. (2022) [22,23], where phenolic compounds neutralized free radicals through hydrogen transfer (HAT), generating phenoxyl radicals that act as free radical scavengers in asphalt matrices [18].

Finally, determined TPC and profiles in both NS aligned with the antioxidant mechanisms proposed by Mousavi et al. (2016) and Sun et al. (2025) [21,49], who demonstrated that lignin-derived phenolic compounds improve high-temperature rheological properties and delay oxidative aging through dual-protection mechanisms, with the following factors: (1) less reactive molecular species that show little propensity toward oxidation, and (2) highly reactive components (such as phenolic hydroxyl groups) that serve as primary targets for oxidative attacks, acting as sacrificing elements to preserve key asphalt components from oxidative degradation.

### 4.2. Low- and High-Temperature Effects

The Fraass fragility point determination revealed highly significant effects (*p* < 0.0001) of HS and WS modification on low-temperature performance throughout all aging stages. The incorporation of the 3% HS modifier significantly improved low-temperature flexibility, reducing the Fraass breaking point from −8.5 °C (control) to −12.3 °C across all aging periods, representing an enhancement of 3.8 °C. These results exceed typical Fraass point improvements reported for conventional polymer-modified asphalts [50] and align with low-temperature performance specifications established in Chilean MC 8.302.24 methodology and international standards such as AASHTO T 313 [51] for bending beam rheometer (BBR) tests. The low-temperature enhancement mechanism may be attributed to the plasticizing effect generated by the lignocellulosic components of nutshells, particularly the holocellulose fraction (46.6%), which can reduce asphalt stiffness at low temperatures [52,53]. At 1% addition, both HS and WS modifiers achieved Fraass temperatures of −7.0 °C and −7.1 °C in control conditions, compared to −4.8 and −4.9 °C for the unmodified binder, demonstrating immediate low-temperature performance improvement. This enhancement was partially maintained after long-term PAV aging, where binders modified with 1% HS and WS exhibited Fraass temperatures of −2.8 and −2.5 °C versus −3.3 °C and −3.1 °C for the control. The progressive deterioration of low-temperature properties with aging is consistent with the thermal stress development mechanisms described in AASHTO TP10 thermal stress restrained specimen test (TSRST) protocols and correlates with the carbonyl formation and molecular weight increase documented by Lu et al. (2021) and Bruneau et al. (2023) [5,7] during oxidative aging processes. However, the limited correlation between Fraass breaking point and other low-temperature parameters (such as BBR stiffness modulus S and m-value) observed in similar studies [54] suggests that the Fraass test, while useful as a screening tool in MC 8.302.24 Chilean specifications, should be complemented with fracture mechanics-based tests to comprehensively characterize low-temperature cracking resistance, particularly for modified asphalts where polymer–asphalt interactions generate complex viscoelastic behaviors not fully captured by empirical breaking point methodologies.

### 4.3. Rutting Performance

The G*/sin δ parameter evaluation at 76 °C revealed a complex interaction between nutshell modification percentage and aging stage, with highly significant effects (*p* < 0.001) determined between nutshell material (N) and incorporation percentage (I%). Under control (no aging) conditions, concentrations of 1 and 3% produced significant reductions in the rutting parameter; HS 1% exhibited a decrease of 19.8% while HS 3% showed a reduction of 20.2% compared to the addition control (0%), with WS displaying similar trends (18.9% and 22.1% reductions for 1% and 3%, respectively). These reductions contradict the minimum G*/sin δ requirement of 1.00 kPa for original binders and 2.20 kPa for RTFOT-aged binders established in AASHTO and Chilean specifications for performance-graded asphalts, suggesting that nutshell incorporation at low percentages may compromise high-temperature rutting resistance in unaged conditions. This negative effect on rutting performance may be attributed to the initial plasticizing effect generated by the lignocellulosic components of nutshells, which reduce binder stiffness through physical dilution mechanisms rather than chemical modification [55,56]. The microstructural characteristics of nutshells, particularly their heterogeneous shape and porous surface [25,31], may create stress concentration points within the asphalt matrix that reduce overall structural integrity under high-temperature shear conditions. This hypothesis is supported by the particle size effect (10–12 μm) employed in this study, which may not provide optimal dispersion and interfacial adhesion within the asphalt binder matrix. Nevertheless, after short-term RTFOT aging, the 3% addition treatments (both HS and WS) exhibited non-significant differences compared to the short-term control sample, indicating that the antioxidant protective effect begins to manifest during thermo-oxidative exposure. This transition aligns with the antioxidant activation mechanisms described by Adwani et al. (2023) and Kong et al. (2024) [15,19], where phenolic compounds become reactive toward free radicals generated during elevated-temperature exposure, progressively mitigating oxidative hardening that would otherwise increase G*/sin δ values beyond optimal performance ranges.

After long-term PAV aging, all treatments exhibited moderate to large increases in G*/sin δ (1.4–5.5%) compared to their respective controls, suggesting limited antioxidant efficacy under prolonged thermo-oxidative stress conditions (100 °C, 20 h, 2.07 MPa air pressure, AASHTO R-28 [42] and EN 14769 [57]). This behavior contrasts with the superior aging resistance reported for lignin-modified asphalts [21,58], indicating that raw nutshell powder may require chemical activation or extraction of concentrated phenolic fractions to achieve comparable antioxidant performance. The formation of carbonyl and sulfoxide functional groups during PAV aging, monitored through FTIR spectroscopy at wavenumbers 1700 and 1030 cm^−1^ respectively per standard protocols [50,59], should be quantified to establish direct correlations between chemical oxidation indices and rheological degradation parameters.

### 4.4. Viscosity-Based Relative Aging Index (RAI)

The viscosity determinations at 60 °C (59–61 °C average) demonstrated significant reductions (*p* < 0.005) with the addition of both nutshells at control aging time, confirming an initial plasticizing effect consistent with the incorporation of lignocellulosic materials into petroleum-based matrices. The Relative Aging Index (RAI) analysis revealed distinguishable patterns across aging stages, with highly significant main effects of nutshell type (*p* < 0.0001) and aging stage (*p* < 0.001), alongside significant interaction effects (*p* < 0.001) indicating differential behavior against oxidative hardening.

After short-term aging (control to short-term transition), RAI values showed a tendency to increase significantly after 1% addition (11.3%) and 2% addition (42.9%) compared to the control, suggesting accelerated oxidative hardening at intermediate modification levels. This paradoxical increase may be attributed to microstructural interference generated by nutshell particles, which could enhance oxygen diffusion pathways within the asphalt matrix during RTFOT exposure (163 °C, 85 min per AASHTO T-240 [41]), exacerbating oxidative reactions rather than mitigating them. Similar phenomena have been reported by Mirwald et al. (2020) and Pipintakos et al. (2020) [60,61], who documented heterogeneous oxidation patterns in modified asphalts where particulate additives create localized oxidation microenvironments.

However, the 3% addition showed a drastic decrease in RAI compared to 2% treatments and exhibited only a 7.7% increase compared to the control, suggesting a threshold concentration effect where sufficient phenolic content becomes available to effectively scavenge free radicals generated during short-term aging. This concentration-dependent antioxidant activity aligns with dose–response relationships, documented by Ouyang et al. (2006) and Apeagyei (2011) [62,63,64], for synthetic antioxidants such as zinc dialkyl dithiophosphate (ZDDP) and Vitamin E in asphalt binders.

Regarding the long-term aging regime (control to PAV), RAI values demonstrated significant decreases for both HS and WS at all modification levels, 1% (10.8–11.3% reduction), 2% (6.2–7.0% reduction), and 3% (14.0–14.3% reduction), compared to the control. These results confirmed the superior long-term antioxidant performance of nutshell modifiers, particularly at 3% concentration, where the cumulative phenolic content may provide sustained free radical scavenging capacity throughout prolonged thermo-oxidative exposure. The aging resistance mechanisms may involve both primary antioxidant effects (direct hydrogen donation to alkyl and peroxyl radicals) and secondary stabilization effects (decomposition of hydroperoxides to non-radical products), as described in antioxidant theory applied to petroleum-derived materials [6,12].

Finally, RAI value, based on rotational viscosity measurements per MC 8.302.15 (LNV41) Chilean specifications and ASTM D4402 standards [65], provides a practical industrial quality control parameter that correlates with more sophisticated rheological measurements. However, viscosity-based aging indexes should be complemented with SARA (Saturates, Aromatics, Resins, Asphaltenes) fractionation analysis by ASTM D4124 [66] to directly quantify compositional changes during aging, particularly asphaltene formation and aromatic depletion, which constitute the fundamental chemical transformations responsible for age-hardening [67,68].

### 4.5. Multiple Stress Creep Recovery (MSCR)

The MSCR analysis conducted per the AASHTO T-350 standard [44] revealed highly significant effects (*p* < 0.001) for nutshell type (N), concentration (I%), and stress level (kPa), with significant interactions (*p* < 0.005) between all factors, demonstrating complex viscoelastic recovery behavior dependent on modification level and aging state. At 0.1 kPa stress level (representative of moderate traffic loading by AASHTO M 332 [69], unaged control samples exhibited 1.56% recovery, while 1% treatments decreased recovery by 46.0–48.1% for HS and WS, respectively, indicating substantial loss of elastic response at low modification levels. This recovery reduction suggests that nutshell particles may interfere with the asphalt colloidal structure, disrupting the asphaltene-resin equilibrium that led to the elastic recovery mechanisms [70,71]. Conversely, 2% treatments showed intermediate recovery values 17.8–23% lower than the control, while 3% treatments exhibited the greatest recovery loss (69.8–71.1% reduction), presenting a concentration-dependent deterioration of elastic response in unaged conditions. This trend contradicts the recovery enhancement typically observed with elastomeric polymer modification [56] and suggests a fundamental incompatibility between rigid lignocellulosic particles and the viscoelastic requirements for high recovery performance. Nevertheless, after short-term RTFOT aging at 0.1 kPa, recovery percentages increased for 1% and 2% treatments (18.8–20% and 12.7–13.1% increases respectively), indicating that thermo-oxidative hardening partially compensates for the initial plasticizing effect, restoring elastic response through increased molecular association and crosslinking. This recovery-enhancement mechanism differs from the oxidative aging deterioration typically observed in unmodified asphalts [72,73,74] and suggests that nutshell phenolic compounds may promote controlled oxidative crosslinking that enhances elasticity without excessive hardening.

At elevated stress levels (3.2 kPa), representing severe loading conditions equivalent to AASHTO M 332 [69] “Extreme” (E) traffic grade (>30 million ESALs), recovery values demonstrated different patterns. Short-term-aged samples with 1 and 2% additions exhibited recovery increases of 31.5–37.3%, while the 3% addition showed decreases of 8.3–12.6% compared to the control, suggesting stress sensitivity threshold effects in which higher NS concentrations reduce recovery performance under extreme loading conditions. These findings align with the stress-dependent behavior documented in MSCR implementation guidance [75], where polymer-modified binders exhibit superior recovery at low stress but may show stress sensitivity at elevated shear stresses.

On the other hand, the recovery difference parameter (R diff%), which quantifies stress sensitivity per AASHTO M 332 specifications (maximum 0.75 permitted), demonstrated decreasing trends with nutshell modification at 1 and 2% levels (2.2–14.4% reduction), indicating improved stress-independent behavior. Lower R diff% values are desirable as they indicate consistent performance across traffic loading spectra, a critical requirement for high-performance asphalts subjected to variable traffic conditions.

The non-recoverable compliance parameter (J_nr_) at 0.1 kPa revealed contrasting behaviors across aging stages. At the control time, 1 and 3% treatments showed significantly higher J_nr_ values (*p* < 0.001), reflected in increases of 20.6–23.3% for 1% and 23.5–27.1% for 3%, indicating greater susceptibility to permanent deformation in unaged conditions. However, after short-term aging, 1 and 2% treatments decreased J_nr_ by 18.1–22.7%, suggesting improved rutting resistance through beneficial oxidative crosslinking. This transition aligns with the rutting performance specifications established in AASHTO M 332, which sets maximum J_nr_, 3.2 kPa values ranging from 4.0 kPa^−1^ for standard traffic (S grade) to 0.5 kPa^−1^ for extremely high traffic (E grade). The determination of whether modified samples meet these traffic-specific criteria requires quantitative J_nr_ reporting at a 3.2 kPa stress level, which should be compared against Chilean traffic loading classifications and regional pavement temperature data to establish appropriate performance grade (PG) designations per MC specifications and the AASHTO M 320 framework. The J_nr_ difference percentage (J_nr_ diff %) results exhibited significant decreases across all aging stages for 1, 2, and 3% treatments compared to the control, indicating superior stress-independent behavior and reduced susceptibility to stress-induced permanent deformation. This parameter directly correlates with polymer modification quality following the AASHTO M 332 criteria, where maximum J_nr_ diff% of 75% is permitted. The systematic reduction in J_nr_ diff % suggests that NS phenolic compounds may generate polymer-like stabilization effects through hydrogen bonding interactions with asphalt polar fractions, improving stress distribution mechanisms similar to those observed in SBS-modified asphalts [73,76].

Finally, MSCR results indicate that nutshell modification generates concentration-dependent and aging-dependent effects on viscoelastic recovery and permanent deformation resistance. While low concentrations (1–2%) show promise for improving aging resistance without catastrophic loss of recovery performance, the 3% addition presents trade-offs between antioxidant protection and elastic response that require optimization through co-modification strategies with elastomeric polymers or chemical activation of nutshell phenolic fractions to enhance compatibility with asphalt matrices [77,78].

## 5. Conclusions

The findings of this study indicate that HS and WS exhibit limited potential as antioxidant additives for asphalt binders, with their effects being strongly dependent on concentration and aging conditions. In addition, HS modifiers consistently outperformed WS across all evaluated parameters. The main effects achieved statistical significance for modifier type in all rheological and performance assessments, supporting the superior effectiveness of hazelnut-shell-derived antioxidant modifiers for asphalt binder enhancement and aging resistance improvement.

The 3% addition treatment (both HS and WS) exhibited the best balance between plasticizing effects and antioxidant protection, preserving rheological properties during prolonged aging and improving thermal flexibility according to the Fraass index. However, significant increases in non-recoverable compliance (J_nr_) present limitations for applications requiring high rutting resistance. The antioxidant mechanisms observed were more effective during thermal aging than thermo-oxidative aging, suggesting that the phenolic compounds in the shells require chemical optimization or co-formulation with secondary antioxidants to maximize their effectiveness.

Future studies should focus on the extraction and purification of specific phenolic fractions, evaluation of synergies with synthetic antioxidants, and detailed molecular characterization of protection mechanisms during different accelerated aging modalities.

Finally, it was demonstrated that forced aging critically impacts the diversity and correlation of key variables that describe the quality and performance of AB, validating the need to consider both short- and long-term effects when designing improved and sustainable materials for industrial applications.

## Figures and Tables

**Table 1:** Commercial CA-24 asphalt binder parameters and properties (DYNAL Industria S.A—Santiago, Chile).

Parameter	Unit	Range/Value	Standard Method [39]
Rotational viscosity 60 °C	Poise (P)	≥2400	8.302.15 (LNV41)
Ductility 25 °C	cm min^−1^	100, min	8.302.8 (LNV35)
Spot test	Xilol %	30% max	8.302.7 (LNV25)
Ignition point	°C	232 min	8.302.9 (LNV36)
Penetration index	Index	−2.0 + 1.0	8.302.21 (LNV48)

## Data Availability

The original contributions presented in this study are included in the article. Further inquiries can be directed to the corresponding author.
